# Retraction

**DOI:** 10.1002/cam4.6713

**Published:** 2023-12-18

## Abstract

‘Interference of the long noncoding RNA CDKN2B‐AS1 upregulates miR‐181a‐5p/TGFβI axis to restrain the metastasis and promote apoptosis and senescence of cervical cancer cells’, by Lihong Zhu, Quanhua Zhang, Shaoping Li, Shan Jiang, Jingjing Cui, Ge Dang, Cancer Med. 2019; 8:1721‐1730. The above article, published online on 18 March 2019 in Wiley Online Library (https://onlinelibrary.wiley.com/doi/full/10.1002/cam4.2040) has been retracted by agreement between the journal's Editor in Chief, Stephen Tait, and John Wiley & Sons Ltd.

The retraction has been agreed following an investigation based on allegations raised by third parties. Multiple image elements in figures 3, 5, and 7 were found to have been published elsewhere in a different scientific context. Thus, the editors consider the conclusions of this article to be invalid. The authors did not respond when asked to collaborate during the investigation.

‘Interference of the long noncoding RNA CDKN2B‐AS1 upregulates miR‐181a‐5p/TGFβI axis to restrain the metastasis and promote apoptosis and senescence of cervical cancer cells’, by Lihong Zhu, Quanhua Zhang, Shaoping Li, Shan Jiang, Jingjing Cui, Ge Dang, Cancer Med. 2019; 8:1721‐1730. The above article, published online on 18 March 2019 in Wiley Online Library (https://onlinelibrary.wiley.com/doi/full/10.1002/cam4.2040) has been retracted by agreement between the journal's Editor in Chief, Stephen Tait, and John Wiley & Sons Ltd.

The retraction has been agreed following an investigation based on allegations raised by third parties. Multiple image elements in figures 3, 5, and 7 were found to have been published elsewhere in a different scientific context. Thus, the editors consider the conclusions of this article to be invalid. The authors did not respond when asked to collaborate during the investigation.

